# Multiplex 16S rRNA‐derived geno‐biochip for detection of 16 bacterial pathogens from contaminated foods

**DOI:** 10.1002/biot.201600043

**Published:** 2016-09-06

**Authors:** Hwa Hui Shin, Byeong Hee Hwang, Hyung Joon Cha

**Affiliations:** ^1^ Department of Chemical EngineeringPohang University of Science and TechnologyPohangKorea; ^2^ Division of BioengineeringIncheon National UniversityIncheonKorea

**Keywords:** Direct RNA‐detection, Foodborne pathogen, Food matrix, Multiple detection, Oligonucleotide microarray

## Abstract

Foodborne diseases caused by various pathogenic bacteria occur worldwide. To prevent foodborne diseases and minimize their impacts, it is important to inspect contaminated foods and specifically detect many types of pathogenic bacteria. Several DNA oligonucleotide biochips based on 16S rRNA have been investigated to detect bacteria; however, a mode of detection that can be used to detect diverse pathogenic strains and to examine the safety of food matrixes is still needed. In the present work, a 16S rRNA gene‐derived geno‐biochip detection system was developed after screening DNA oligonucleotide specific capture probes, and it was validated for multiple detection of 16 pathogenic strains that frequently occur with a signature pattern. rRNAs were also used as detection targets directly obtained from cell lysates without any purification and amplification steps in the bacterial cells separated from 8 food matrixes by simple pretreatments. Thus, the developed 16S rRNA‐derived geno‐biochip can be successfully used for the rapid and multiple detection of the 16 pathogenic bacteria frequently isolated from contaminated foods that are important for food safety.

**Abbreviations**
PCR: polymerase chain reaction
RDP: Ribosomal Database Project

## 1 Introduction

Foodborne diseases occur worldwide. Approximately 300 cases of outbreaks involving more than 6000 patients annually are reported in Korea 1, 2. Mild symptoms include diarrhea, nausea, vomiting, and fever 3. However, severe symptoms, such as sepsis, hemolytic uremic syndrome, and/or death, sometimes arise in certain patients, such as infants, older adults, or immunocompromised patients 4, 5. Foodborne diseases are mainly caused by various pathogenic bacteria, including *Campylobacter* spp., *Shigella* spp., *Salmonella* spp., and pathogenic *Escherichia coli* 3, 5. Therefore, to prevent foodborne diseases and minimize the impact of foodborne outbreaks caused by contaminated foods and agricultural products, it is important to inspect contaminated foods and to detect diverse pathogens accurately and simultaneously.

For this reason, a large number of detection and diagnosis methods have been developed. Culture‐based conventional methods are commonly used to detect bacteria, but they are time‐consuming, labor‐intensive, and difficult to quantitatively analyze 6, 7. Therefore, diverse rapid technologies have been devised from molecular‐based methods such as polymerase chain reaction (PCR) 8, 9, 10, 11, 12, in situ hybridization assays 13, 14, and microarrays 15, 16, 17, 18, 19. In particular, the DNA microarray is considered to be a potent device that allows multiple, specific, and sensitive screening for pathogen identification. DNA oligonucleotide probes with a short length (≈20–80 bp) in arrays can improve the accuracy and reproducibility of results by increasing specificity and standardizing the hybridization process compared to longer DNA probes 20. Several DNA oligonucleotide biochips based on 16S rRNA information have been investigated to detect bacteria, and 16S rRNA containing conserved and variable sequences has been widely used for phylogenetic discrimination 4, 21, 22, 23. Previously, we developed the 16S rRNA‐based microarrays for detection of seven pathogens from pure culture 16 and 11 pathogens with pattern‐mapping 18. In addition, the detection technique was developed with 16S rRNA targets 19. However, a detection method that can be used to identify more diverse pathogenic strains and can be used to check the safety of food matrixes is still necessary.

In the present work, a 16S rRNA‐derived geno‐biochip system was developed for the multiple detection of 16 pathogenic bacteria (*Bacillus cereus*,* Campylobacter jejuni*, *Clostridium perfringens*, *Escherichia coli*, *Escherichia coli* O157:H7, *Listeria monocytogenes*, *Salmonella enterica* subsp. *enterica* serotype Choleraesuis, *Salmonella enterica* subsp. *enterica* serotype Enteritidis, *Shigella boydii*, *Shigella dysenteriae*, *Staphylococcus aureus*, *Vibrio cholerae*, *Vibrio parahaemolyticus, Vibrio vulnificus,* and* Yersinia enterocolitica*), which frequently occurred worldwide in agricultural products 1, 24, using rRNAs as detection targets directly from cell lysates without any purification and/or amplification process. In addition, a simple pretreatment method was employed to separate bacterial cells from various food matrixes.

## 2 Materials and methods

### 2.1 Pathogenic strains

For the design of specific capture probes, 23 strains of 16 microbial species were used (Supporting information, Table S1): *B. cereus* (American Type Culture Collection (ATCC; Manassas, VA, USA) 11778, 13061, and 14579), *C. jejuni* (ATCC 33291), *C. perfringens* (ATCC 13124), *E. coli* (ATCC 25922), *E. coli* O157:H7 (ATCC 43894), *L. monocytogenes* (ATCC 15313), *S. boydii* (ATCC 8700 and 35966), *S. dysenteriae* (ATCC 13313), *S. sonnei* (ATCC 9290, 25931, and 29930), *S. aureus* (ATCC 6538), *S. enterica* subsp.* enterica* serotype Choleraesuis (ATCC 7001, 10708, and 13312), *S. enterica* subsp. *enterica* serotype Enteritidis (ATCC 31194), *V. cholerae* (ATCC 14035),* V. parahaemolyticus* (ATCC 17802), *V. vulnificus* (ATCC 27562), and *Y. enterocolitica* (ATCC 23751).

### 2.2 Design and synthesis of DNA oligonucleotide specific capture probes

16S rRNA sequences of all bacterial strains were acquired from the Ribosomal Database Project (RDP) II database (Centers for Microbial Ecology, East Lansing, MI, USA) or the National Center for Biotechnology Information (NCBI, http://www.ncbi.nlm.nih.gov/gene) Genbank. Their sequences were also directly obtained by sequencing polymerase chain reaction (PCR) amplicons to compare them with those from databases (data not shown). Because 16S rRNA sequences show high similarity between closely related species, the closest comparison method was employed, that is, species‐specific regions were checked by the alignment of the most similar two 16S rRNA sequences using BioEdit software (Ibis Biosciences, Carlsbad, CA, USA). Next, these chosen regions were compared to all 16S rRNA sequences of target and target‐related species. Based on sequence dissimilarities of over 10–15% compared to other species sequences, capture probe candidates were designed with similar melting temperatures using Primer Premier 5 (Premier Biosoft International, Palo Alto, CA, USA). Finally, capture probe candidates were searched using the RDP II database and NCBI Blast searches to confirm their similarities with other bacteria. Consequently, new 12 specific capture probe candidates of five species were designed based on the above criteria (Supporting information, Table S2).

Next, the designed DNA oligonucleotide capture probe candidates were chemically synthesized with 5'‐end modification (Integrated DNA Technology, IA, USA). After the experimental selection of the capture probe candidates (Supporting information, Fig. S1 and S2, Table S3), seven specific capture probes were ultimately selected from the newly designed candidates. A total of 27 specific capture probes including 20 previously designed capture probes 16 (Table 1), were used to discriminate 15 important pathogenic species. Positive control and artificial standard capture probes 18, 19, 25 were also employed (Table 1).

**Table 1:** S rRNA–derived DNA oligonucleotide capture probes used in this study and their thermodynamic propertiesa)

Bacteria	Probe name	Sequences (5'‐3', 5'‐amine‐spacer, spacer: C6)	Length (bp)	*T*_m_ (°C)	Rating	Reference
All bacteria (positive control)	POCO	GCCGCCAGCGTTCAATCTGA	20	66.5	91	16
*Bacillus cereus*	BACE‐1	CCTCGCGGTCTTGCAGCTCTT	20	63	81	this study
	BACE‐2	CCACCTGTCACTCTGCTCCCG	21	65	100	this study
*Campylobacter jejuni*	CAJE‐1	CACTCTAGACTATCAGTTTCCCAAGC	26	68.6	88	this study
	CAJE‐2	TACCCCTACACCACCAATTCCATCTG	26	67.3	91	this study
*Clostridium perfringens*	CLPE	CGGAGGTGTTGAAACCCCCA	20	65	100	this study
*Escherichia coli*	ESCO	GAAGGCACATTCTCATCTCTGAAAAC	26	62.9	91	16
*Escherichia coli* O157:H7	ESCOO‐1	CAGCAAAGAAGCAAGCTTCTTCCT	24	63.8	73	16
	ESCOO‐2	ACTCGTCAGCAAAGAAGCAAGCT	23	62.7	86	16
*Listeria monocytogenes*	LIMO‐1	GCATGCGCCACACTTTATCATT	22	62.6	80	16
	LIMO‐2	CCATCTTTCAAAAGCGTGGCAT	22	64.0	90	16
*Salmonella enterica* subsp. *enterica* serotype Cholerasuis	SACH‐1	TGCTGCGGTTATTAACCACAACA	23	63.2	86	16
	SACH‐2	GACTCAAGCCTGCCAGTTTCGA	22	64.2	87	16
*Salmonella enterica* subsp. *enterica* serotype Enteritidis	SAEN	AGGCACAAATCCATCTCTGGATTC	24	63.5	74	16
*Shigella boydii*	SHBO‐1	CCCCACCAACAAGCTAATCCC	21	63.1	57.1	this study
	SHBO‐2	ACATTCTCATCTCTGAAAACTTCCGT	26	62.1	92	this study
*Shigella dysenteriae*	SHDY‐1	AGGCACCCTCGTATCTCTACAAGG	24	63.1	89	16
	SHDY‐2	CCGCCACTCGTCAGCAAAGCA	21	68.9	100	14
*Staphylococcus aureus*	STAU‐1	AACTAGCTAATGCAGCGCGGAT	22	63.4	81	16
	STAU‐2	AGATGTGCACAGTTACTTACACATATGTTCT	31	63.0	74	16
*Vibrio cholera*	VICH‐1	CCTCTACCGGGCAATTTCC	19	63.5	79	16
	VICH‐2	CTCTACCGGGCAATTTCCCA	20	62.3	79	16
*Vibrio parahaemolyticus*	VIPA‐1	CCCGAAGGTTCAGATAACTCGTTT	24	63.1	88	16
	VIPA‐2	CGTTATCGTTCCCCGAAGTTCAGAT	25	66.9	93	14
*Vibrio vulnificus*	VIVU‐1	AAACAAGTTTCTCTGTGCTGCCGC	24	66.9	91	14
	VIVU‐2	TGAGCCGAAGCTATCATGCGG	21	65.8	83	14
*Yersinia enterocolitica*	YEEN‐1	GTTATTGGCCTTCCTCCTCGCT	22	63.8	84	16
	YEEN‐2	TGCGAGTAACGTCAATCCAACAA	23	63.1	89	16
Artificial standard	ARST	CCCAAGGGAACCCAAGGGAAA	21	66.8	85	23
Artificial standard target	ARSTT	TTTCCCTTGGGTTCCCTTGGG‐Alexa flour 647	21	66.8	85	23

a) ll thermodynamic properties were calculated by Primer Premier.

### 2.3 Design of the geno‐biochip format

**Figure 1:**
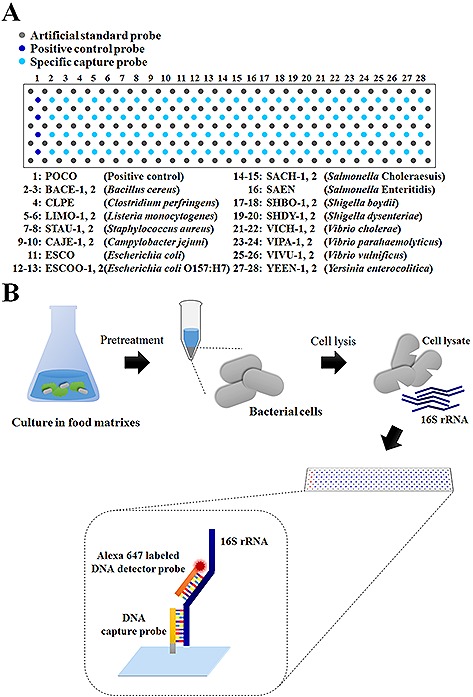
Schematic diagrams of (**A**) the array format for the 16S rRNA‐derived geno‐biochip containing 27 specific capture probes and (**B**) the schematic steps of the 16S rRNA detection of bacterial cells directly separated from food matrixes.

The DNA geno‐biochip was prepared with a format in which each specific capture probe was surrounded by four rectangular‐shaped spots of artificial standard capture probes (Fig. 1A). The specific capture probes were immobilized as four‐repeated spots. The array used for the selection of capture probes contained 34 × 5 spots of the artificial standard capture probe, 1 × 4 spots of the positive control capture probe on the first line, and 1 × 4 spots of each specific capture probe (to include a total of 32 probes) (Supporting information, Fig. S1). After probe selection, the final geno‐biochip contained 29 × 5 spots of the artificial standard capture probe, 1 × 4 spots of the positive control capture probe on the first line, and 1 × 4 spots of each specific capture probe (to include a total of 27 probes) (Fig. 1A).

### 2.4 Preparation of the geno‐biochip

The geno‐biochip was fabricated following previously described procedures 16, 18, 19. Specifically, NH_2_‐modified DNA oligonucleotide probes were spotted on aldehyde‐coated slides (Super Aldehyde; Telechem International, Sunnyvale, CA, USA). Each DNA oligonucleotide (10 μM) was dissolved in a 3x SSC spotting buffer solution (450 mM NaCl, 3 mM tri‐sodium citrate, and *N*,*N*,*N*‐trimethyl glycine (betaine; Sigma); pH 6.6, final concentration 1.5 M). The oligonucleotide capture probes were printed on the slides using a Microsys 5100 microarrayer (Cartesian Technologies, Ann Arbor, MI, USA) with the Chip Maker 2 pin (Telechem International) at 74% humidity in a class 10 000 clean room. After spotting and overnight incubation in low (≈30%) humidity conditions, the geno‐biochip slides were dipped in a solution containing 1.3 g of NaBH_4_ dissolved in 375 mL of phosphate‐buffered saline (PBS; pH 7.4) and 125 mL of ethanol for 5 min, followed by washing twice in 0.2% sodium dodecyl sulfate (SDS) for 1 min each, and twice with distilled water (DW). Slides were dried by centrifugation at 1500 rpm for 3 min, and stored at room temperature under a vacuum until further use.

### 2.5 Genomic DNA isolation and 16S rDNA target preparation

All species were cultured in nutrient broth (Difco, Kansas, MO, USA) at 30 or 37°C, except for *V. cholerae*, *V. parahaemolyticus*, and *V. vulnificus*, which were cultured in trypticase soy broth (TSB; Difco) with 2% NaCl at 37°C and *C. perfringens*, which was cultured in reinforced clostridial medium (Difco) at anaerobic 37°C. The strains of *E. coli* O157:H7 and *C. jejuni* were acquired as purified genomic DNAs. Each genomic DNA was extracted and purified using DNeasy^®^ Tissue Kit (Qiagen GmbH, Hilden, Germany). Genomic DNA concentration and purity were checked with a UV/vis spectrometer (Shimadzu, Kyoto, Japan).

Each purified genomic DNA was used as a template for the amplification of 16S rDNA using PCR, followed by fluorescence labeling of each DNA target. The universal primer set (forward: 5′AGAGTTTGATCMTGGCTCAG‐3′, backward: 5′TACGGYTACCTTGTTACGACTT‐3′; Genotech, Daejeon, Korea) used in this study includes the first and ninth conserved regions of 16S rDNA 22. The PCR mixture was composed of 1–50 ng/mL of genomic DNA, 2 U *Taq* polymerase (Takara, Otsu, Japan), 2 μM forward universal primer, 2 μM reverse universal primer, 0.5 mM dATP, 0.5 mM dCTP, 0.5 mM dGTP, 0.3 mM dTTP, 0.15 mM amine‐modified dUTP, and 1x *Taq* buffer. PCR was performed in the DNA thermal cycler (BioRad, Hercules, CA, USA) under the following conditions: 95°C for 5 min, 30 cycles of 95°C for 1 min, 60°C for 45 s, and 72°C for 1 min 30 sec, followed by 72°C for 5 min. A Wizard^®^ SV gel and PCR clean‐up system (Promega, Madison, WI, USA) was employed for the purification of amine‐modified amplicons, followed by ethanol precipitation. Next, each purified 16S rDNA amplicon was labeled using an ARES™ Alexa Fluor^®^ 647 DNA labeling kit (Molecular Probes, Eugene, OR, USA) and purified by the PCR clean‐up kit once more before use.

### 2.6 Hybridization and fluorescence intensity scanning

Before hybridization, the fabricated geno‐biochip was pre‐incubated in the buffer containing a 3x SSC solution (450 mM NaCl and 3 mM tri‐sodium citrate; pH 7.0) with 1% w/v bovine serum albumin (BSA; Sigma, St. Louis, MO, USA) and 0.1% w/v SDS for 30 min at 50°C. The array was washed three times (twice with DW and once with ethanol) and dried by centrifugation at 1500 rpm for 3 min. Then, the array was spiked with the hybridization solution (3x SSC, 0.1% w/v SDS, and 0.2% w/v BSA) at 50°C for 1 h containing 50–150 μg/mL PCR‐amplified target 16S rDNA and 1 μM artificial standard target DNA 25. Next, the array was washed four times: the first with buffer I (1x SSC and 0.2% w/v SDS) for 1 min, the second with buffer II (0.1x SSC and 0.2% w/v SDS) for 1 min, and the third and the fourth with buffer III (0.1x SSC) for 1 min at room temperature. After drying, the geno‐biochip was scanned using a commercial confocal laser scanner (ScanArray Lite; GSI Lumonics, Wilmington, MA, USA), and the data were analyzed using quantitative microarray analysis software (QuantArray; GSI Lumonics).

### 2.7 Direct RNA‐detection of pathogen with eight food matrixes

A total of eight foods were tested as the model for each food matrix: rice for the grain matrix, pork for the meat matrix, eggs for the livestock matrix, canned corn for the processed agricultural matrix, fish cakes for the processed seafood matrix, milk and cheese for the processed livestock matrix, and ham for the processed meat matrix. As a model target pathogen, *S.* Enteritidis was cultured in nutrient broth with 12.5% w/v of each food at 37°C for one day. To separate the bacterial cells from food matrixes, each food matrix was pretreated by different procedures. The samples of rice, canned corn, and fish cakes were centrifuged at 500 rpm for 1 min to remove the large volume of solids. Then, each supernatant was collected and recentrifuged at 13 000 rpm for 1 min. The pellet was washed three times using PBS solution. The cheese sample was centrifuged at 13 000 rpm for 1 min to remove the floating matters and the rest of the process was same as previously described. The egg sample was centrifuged at 13 000 rpm for 2 min. Layers of bacterial cells and egg‐food solids were formed, and the layer of bacterial cells was resuspended into the supernatant. After centrifugation at 13 000 rpm for 1 min, the cell pellet was washed three times with PBS. The milk sample, which was a liquid, did not require removal of particles. The meat sample, which could not be separated with bacterial cells, was centrifuged at 13 000 rpm for 1 min. The cell pellet was washed as previously described.

After the pretreatments of food matrixes, each cell pellet was stored in a deep freezer (−80°C) for at least 30 min and was then resuspended in lysis buffer (1 M NaOH, 0.1% Triton X‐100, and 2 mM EDTA in 20 mM Tris‐HCl pH 8.0) with 1 mg mL^−1^ lysozyme for 5 min at room temperature. After incubation, 1 M sodium phosphate buffer (pH 7.2) was added and mixed. Immediately, the prepared cell lysate containing the total RNAs was mixed with the hybridization buffer (4x SSPE (0.6 M NaCl, 40 mM NaH_2_PO_4_, and 4 mM EDTA), 0.4x Denhardt's solution, 30% v/v formamide, 0.2 μM Alexa 647‐labeled artificial standard target 23, 0.2 μM Alexa 647‐labeled detector probe (DP, Universal 2) 19 and hybridized onto the developed 16S rRNA‐derived geno‐biochip at room temperature for 1 h (Fig. 1B). After hybridization, the biochip was washed three times: first with buffer I (1x SSPE and 0.2% v/v Triton X‐100) for 30 s, second with buffer II (0.1x SSPE and 0.2% v/v Triton X‐100) for 30 s, and third with buffer III (0.1x SSPE) for 30 s at room temperature. After drying, the biochip was scanned using a commercial confocal laser scanner, and the data were analyzed using quantitative microarray analysis.

## 3 Results

### 3.1 Design and selection of 16S rRNA‐derived DNA oligonucleotide specific capture probes

Previously, 20 specific capture probes were designed and selected for 10 pathogenic bacteria 18. To design capture probes for the specific detection of five new pathogens (*B. cereus*, *C. jejuni*, *C. perfringens*, *S. boydii*, and *S. sonnei*), all collected 16S rRNA sequences of target bacteria were aligned and analyzed to obtain DNA sequence fragments 20–25 bp in length with over 10–15% dissimilarity. The matching analysis based on the RDP II database and NCBI blast search was performed to confirm the dissimilarity of searched gene fragments between specific bacteria and others. After searching the sequence fragments, their thermodynamic properties were analyzed to select 12 capture probe candidates, which have similar melting temperatures, with formerly designed probes with differences within 2°C (Supporting information, Table S2).

Before the hybridization experiments, an in silico analysis was performed based on sequence comparison between the directly sequenced data and the databases (data not shown). Most sequences of capture probe candidates were the same as those of the respective bacterial genome database. Although some had mutations, these differences were presumed to not significantly affect the hybridization results because their dissimilarities were lower than 10% as the lowest threshold of the specificity. However, SHSO‐1 (the capture probe for *S. sonnei*) showed low dissimilarity (9.5%) with *S. boydii*, indicating that the discrimination of *S. boydii* with *S. sonnei* might be difficult based on the fluorescence signal of SHSO‐1 due to the nonspecific false‐positive signal.

**Figure 2:**
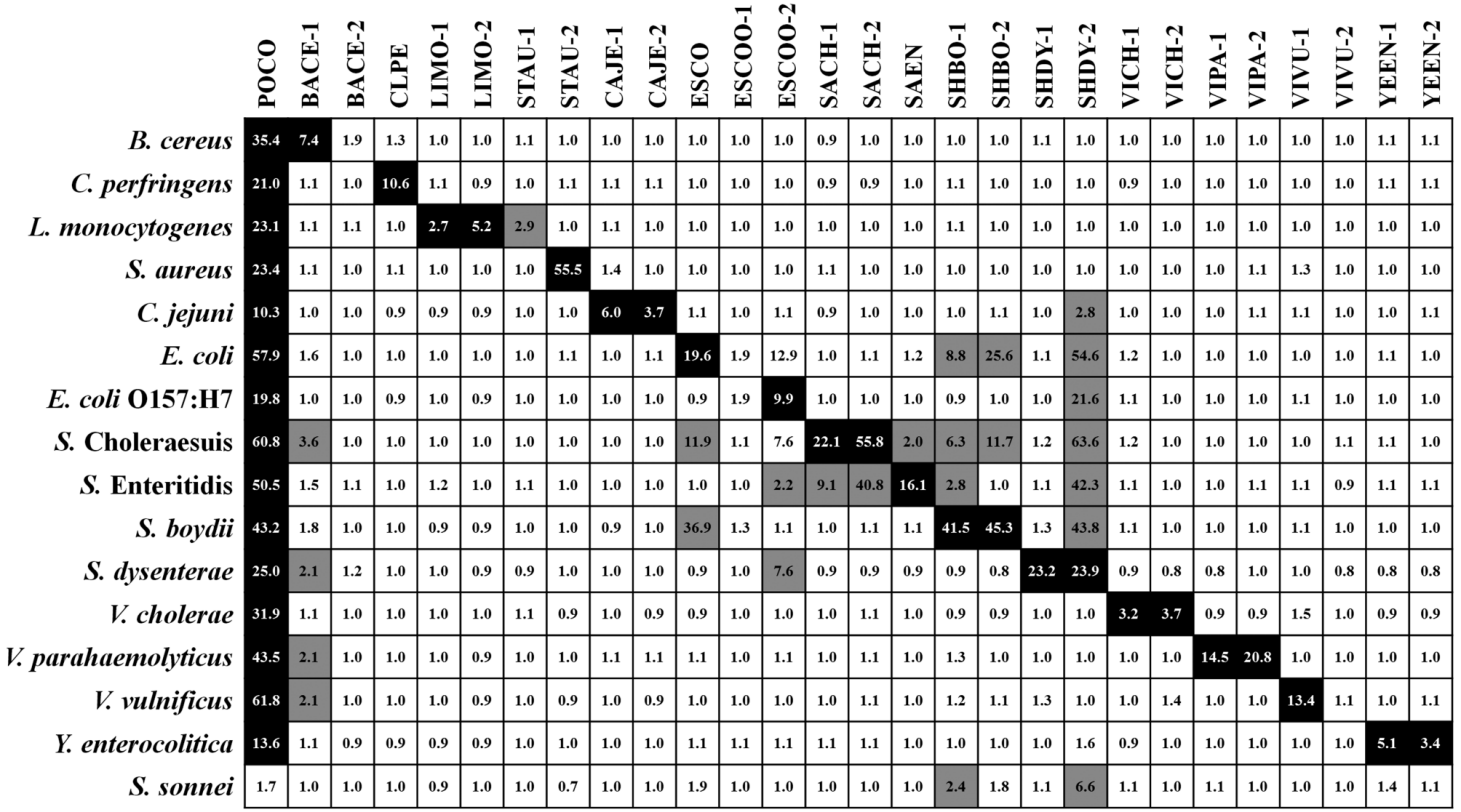
The heat map plot of *S*/*N* of fluorescence intensities acquired from hybridization with each amplified target. Inner numbers indicate the *S*/*N* of each spots. The signals were marked as different colors: black for true‐positive spots; gray for false‐positive spots; and white for negative spots. Positive signals were determined when *S*/*N* was equal to or higher than 2, and negative signals were determined when *S*/*N* was lower than 2.

The detection abilities of newly designed 12 capture probe candidates were examined using hybridization experiments with 20 previously designed capture probes (Table 1). For the experiments, the probe screening geno‐biochip was fabricated to contain a total of 33 capture probes (Supporting information, Fig. S1). 16S rDNA PCR amplicons of six bacteria including *B. cereus*, *C. jejuni*, *C. perfringens*, *S. boydii*, *S. sonnei*, and *S. dysenteriae* were tested to confirm the selectivity and cross‐reactivity of 12 capture probe candidates (Supporting information, Fig. S2). Most of the newly designed specific capture probes showed true‐positive signals with their target bacteria, but three spots of CLPE‐2, SHBO‐3, and SHSO‐2 were false‐negative even though target DNAs were hybridized (Supporting information, Figs. S2B S2D and S2F, Table S3). Unfortunately, BACE‐3 spots were frequently observed as false‐positive signals for every tested pathogenic bacterium (Supporting information, Figs. S2B–F, Table S3). Three *Shigella* spp., including *S. dysenteriae*, were tested to determine the cross‐reactivity among them, and they displayed several false‐positive signals with specific probes of other *Shigella* spp. or *E. coli* (Supporting information, Figs. S2D–F, Table S3). Based on repeated experiments, the three capture probes of false‐negatives (CLPE‐2 and SHBO‐3) and false‐positive (BACE‐3) were excluded. In addition, two capture probes (SHSO‐1 and SHSO‐2) of *S. sonnei* were excluded because they were strongly false‐positive with other target bacteria and ineffective for detection.

### 3.2 Multiple detection of 16 pathogens using amplified target DNA

After the selection of seven specific capture probes for five new pathogens, the complete 16S rRNA‐derived geno‐biochip containing 27 capture probes, including 20 previously designed capture probes was fabricated for the multiple detection of a total of 16 pathogenic bacteria (Fig. 1A). Although the specific capture probes of *S. sonnei* were excluded for final construction of the geno‐biochip, we also conducted its detection because it was expected that *S. sonnei* would be discriminated by different signal pattern from *Shigella* spp. The detected spots of hybridization images (Supporting information, Fig. 3S) were represented by a heat map plot of signal to noise (*S*/*N*) to comprehend detection signals (Fig. 2). Positive spots were determined if *S*/*N* was equal to or higher than 2, and negative spots were determined if *S*/*N* was lower than 2 26.

**Figure 3:**
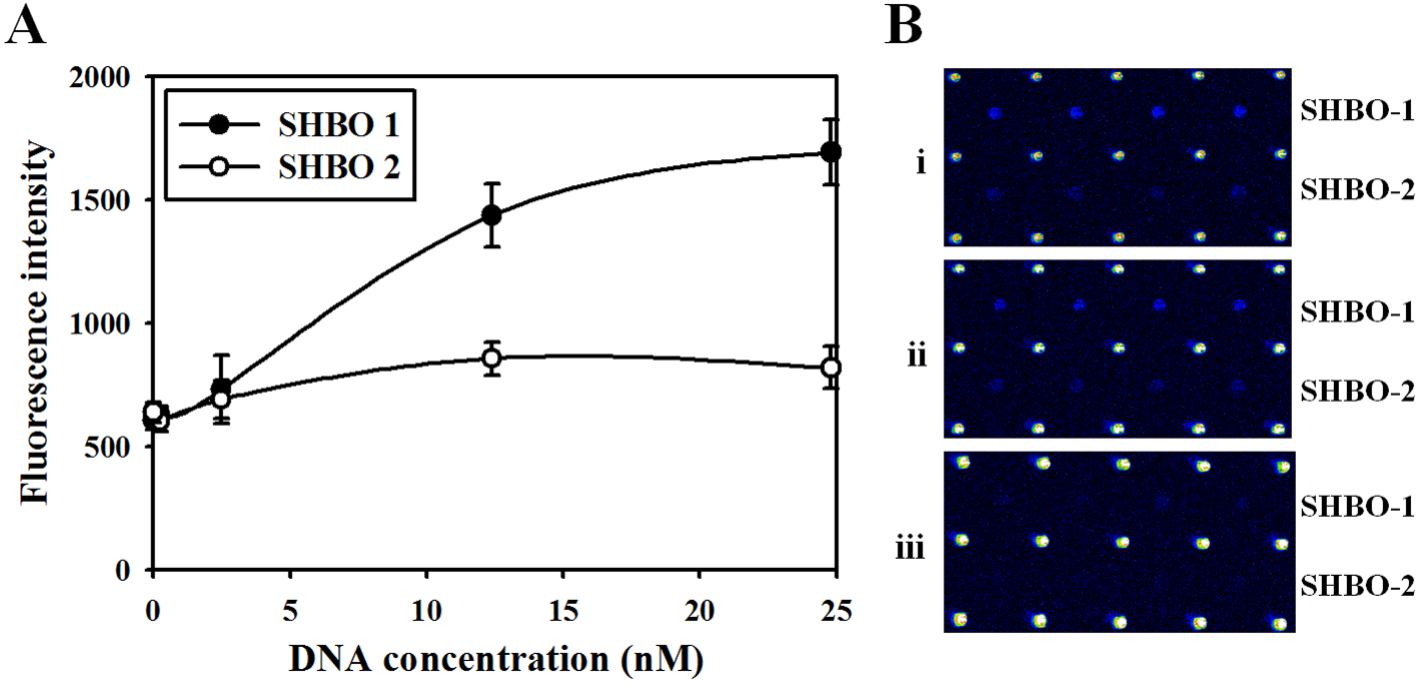
Sensitivity of the 16S rRNA‐derived geno‐biochip. The diluted PCR amplicons of *S. sonnei* were used for sensitivity determination. (**A**) The plot of dynamic detection ranges based on the fluorescence intensities according to target DNA concentration changes for each capture probe SHBO‐1 (closed circle) and SHBO‐2 (open circle). (**B**) Raw hybridization images for (i) 24.8 nM, (ii) 12.4 nM, and (iii) 2.48 nM 16S rDNA targets. Each value of plot was the mean of four repeated spots, and the error bars represent standard deviation.

As a result, 16 target bacteria were successfully discriminated by their specific patterns (Fig. 2). However, similar to previous studies 16, 18, strains of *Shigella*, *Salmonella*, and *E. coli* had false‐positive signals with mutual capture probes. Among the newly designed capture probes, SHBO‐1 and SHBO‐2 showed false‐positive signals with target amplicons of *E. coli* and *S.* Choleraesuis. Although there were some false‐positive spots, the developed 16S rRNA‐derived geno‐biochip containing 27 specific capture probes was able to successfully detect 16 target pathogens based on their signature signal patterns.

### 3.3 Sensitivity of the developed 16S rRNA derived geno‐biochip

The sensitivity of the developed 16S rRNA‐derived geno‐biochip was measured using *S. boydii* (Fig. 3) as a representative strain. The five diluted 16S rDNA amplicons (0–24.8 nM) of *S. boydii* were prepared and applied to the hybridization tests. Then, the dynamic detection ranges were plotted based on the fluorescence intensities according to the concentration changes of target DNA. The fluorescence intensities of both SHBO‐1 and SHBO‐2 were linear below the concentration of 12.4 nM and saturated above that concentration. Both SHBO‐1 and SHBO‐2 signals were not observed at the point of 0.25 nM and the fluorescence intensity was lower or similar with that at the point of 0 nM. Thus, the limit of detection (LOD) was estimated as a range of 0.25–2.5 nM, which was calculated to be 10–100 fmol of the PCR product.

### 3.4 Direct RNA‐detection of pathogen using target RNAs followed by the pretreatment of eight food matrixes

To apply the 16S rRNA‐derived geno‐biochip system for food inspection, it was necessary to develop the pretreatment method for the isolation of bacteria from various complex food matrixes. To test these food matrixes, eight foods were chosen as representative models for each food matrix and bacteria were cultured in medium with each food: rice for a grain matrix, pork for a meat matrix, eggs for a livestock matrix, canned corn for a processed agricultural matrix, fish cakes for a processed seafood matrix, milk and cheese for a processed livestock matrix, and ham for a processed meat matrix. The pretreatment of food matrixes was simply accomplished using centrifugation. In the case of food matrixes with relatively heavy solids (e.g., rice, canned corn, and fish cakes), the bacterial cells were separated from foods using low‐rpm centrifugation. Because milk is a liquid, it did not require removal of the solids and its pretreatment was the simplest. The egg and cheese samples, which retained tiny floating particles, required centrifugation at 13 000 rpm. The bacterial separation was relatively more difficult than for the solid matrixes. In the pork samples, it was impossible to remove the meat particles from bacterial cells just using centrifugation; thus, it was used without pretreatment. As a result, bacterial cells could be obtained from various food matrixes by simple pretreatment using centrifugation.

**Figure 4:**
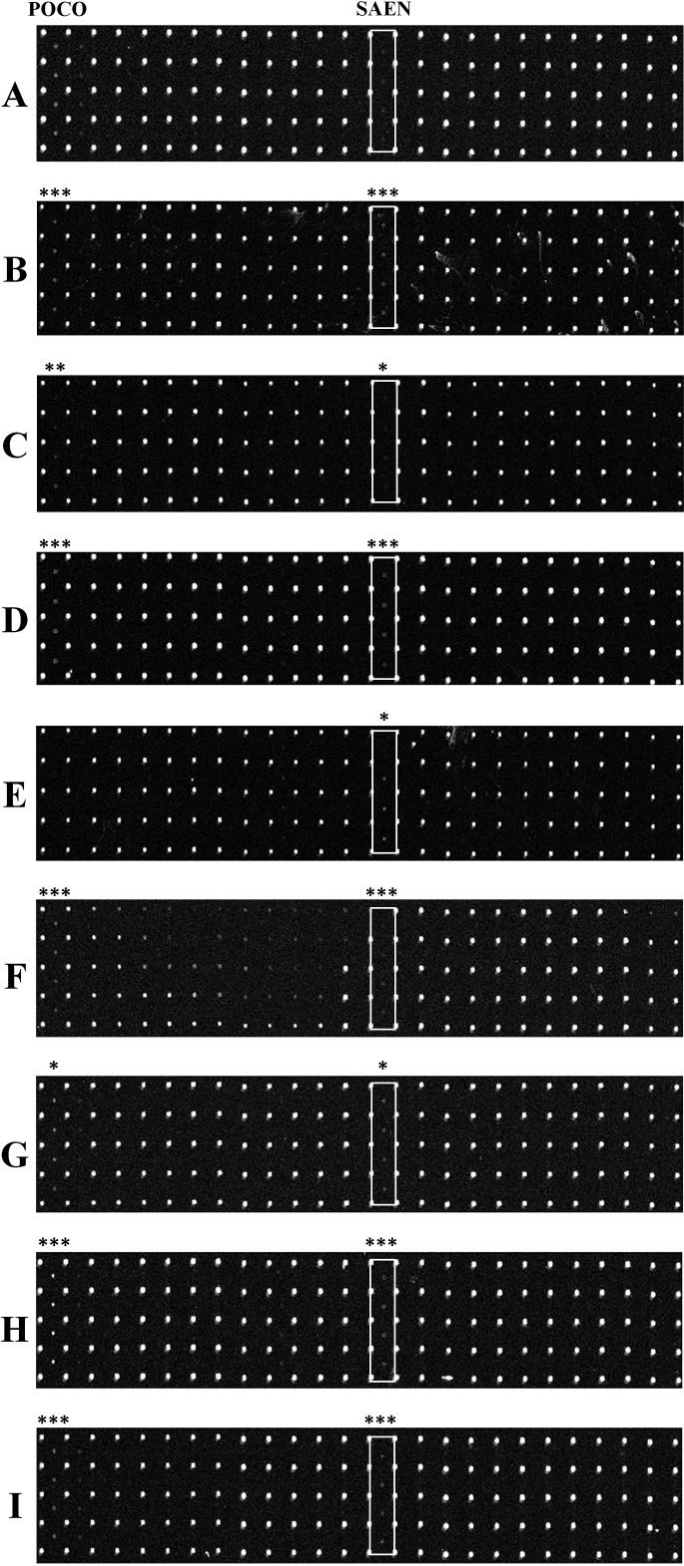
The raw images of hybridization with complete RNA targets directly obtained from isolated S. Enteritidis cells in food matrixes using the rRNA‐derived geno‐biochip in Fig. 1. (**A**) control, (**B**) pork, (**C**) egg, (**D**) milk, (**E**) rice, (**F**) cheese, (**G**) canned corn, (**H**) ham, and (**I**) fish cake. White box indicates specific spots. Asterisks on images represent each p‐value range of POCO and SAEN calculated by paired *t*‐tests. * indicates *p* < 0.05, ** indicates *p* < 0.01, and *** indicates *p* < 0.0005.

After the pretreatment of food matrixes, RNAs containing 16S rRNAs directly obtained from bacterial cells were applied to the geno‐biochip detection system. As the representative target pathogen, *S.* Enteritidis acquired from each food matrix showed specific spots in the hybridization results (Fig. 4). The hybridization results of the pork sample showed the high fluorescence intensity of the background. The fluorescence intensities of specific spots for the egg and cheese samples were also lower than for those of other food samples. Despite these relatively low fluorescence intensities, it was confirmed that pathogens from contaminated food matrixes could be directly detected using our 16S rRNA‐derived geno‐biochip detection system.

## 4 Discussion

In the present work, the multiple detection of 16 target pathogens based on signature signal patterns was successful using our developed 16S rRNA‐derived geno‐biochip containing 27 elaborately designed specific capture probes. The selection of capture probes was based on optimal probe design principles and validation steps of (i) the sequence dissimilarity over 10–15%, (ii) thermodynamic properties, (iii) database matching analysis, (iv) in silico analysis, and (v) probe screening experiments. As a result, capture probes were selected to maximize true‐positive signals and minimize false‐positive signals. Although some false‐positive signals were inevitable due to the high sequence similarity of 16S rRNAs, each bacterium was identified by the characteristic pattern in the rows shown in Fig. 2.

Unexpectedly, some capture probe candidates (CLPE‐2, SHBO‐3, and SHSO‐2) elicited true‐negative signals with their target bacteria. This may have been because the amplified PCR products share the small‐fraction sequences because CLPE‐2 is located near to the end of the amplified 16S rRNA gene. In addition, several factors, such as the secondary structure of PCR amplicons and fragmentation during denaturing steps, might hinder the hybridization between the capture probe and the target DNA 27, 28, 29, 30.

The detection results of three *Shigella* spp. showed several false‐positive signals by the capture probes related to *Salmonella*, other *Shigella* spp. or *E. coli*. These results coincide with the previous study 18. It is well known that *Shigella* spp. are considered to be anaerobic biotypes of *E. coli* and highly‐closed strains 31, 32, 33. Thus, it was quite difficult to strictly discriminate only true‐positive signals of specific probes and eliminate the false‐positive signals among them due to their very analogous 16S rRNA genes 18, 33, 34. Nevertheless, each distinctive signal pattern could identify each strain of *Shigella* spp., *Salmonella* spp., and *E. coli*. For examples, detection results of *E. coli* were different in signals of ESCO, SHBO‐1, and SHBO‐2 compared to signal pattern of *E. coli* O157:H7 (Fig. 2). The signal pattern of *S.* Choleraesuis had differences in signals of ESCO and SHBO‐2 compared to that of *S.* Enteritidis. Three *Shigella* spp. also had all different signal patterns in the probes of ESCO, ESCOO‐1, and SHBO‐1. Consequently, based on the signature pattern according to each bacterium, all pathogenic bacteria could be specifically discriminated with our developed 16S rRNA‐derived geno‐biochip. Indeed, it might need to employ novel biomarkers such as virulence factor genes for more specific detections of *Salmonella*, *Shigella*, and *E. coli*. Previously, we conducted the study for specific discrimination of three *Salmonella* serotypes with a new biomarker gene 35. However, because the use of such biomarker gene is restricted to detection of a particular bacterium, the 16S rRNA‐derived geno‐biochip might be more suitable for multiple detection of various bacterial species in food samples.

The LOD of the developed 16S rRNA‐derived geno‐biochip was estimated using representative *S. boydii* strain in a range of 0.25–2.5 nM, equivalent to 10–100 fmoles of the PCR product. The LOD of 86–172 fmol which was evaluated using the amplified *C. jejuni* 16S rDNA (Supporting information, Fig. S4) was also consistent with the LOD using *S. boydii*. In the previous study, it was reported that the sensitivity of 16S rRNA‐derived geno‐biochip ranged from 6 fmol of the PCR product for *Mycoplasma bovis*, *Streptococcus agalactiae,* and *Streptococcus pyogenes* to 12 fmol for *Staphylococcus aureus* 36. Although the sensitivity of this study was somewhat lower than the previous report using the same detection markers, the specific capture probes designed in this study could also detect targets as low as the fmol level.

Most of the specific signals were detected directly using the developed 16S rRNA‐derived geno‐biochip system with total RNAs containing 16S rRNAs from isolated bacterial cells from contaminated food matrixes. While the use of genomic DNAs as target materials needs a heat‐denaturation step to effectively detect the fluorescence signal 37, the denaturation step was not necessary for RNAs. The detection results using 16S rRNAs showed lower fluorescence intensities than those using amplified 16S rDNAs. This was mainly caused by the inherent unstable characteristics of RNA compared to DNA in experimental environments 38, 39. Therefore, it might be necessary to increase RNA stability during test by employing several strategies such as addition of RNase inhibitor, use of RNA stabilization solution, lowering hybridization temperature, and optimization of magnesium ion concentration which were reported to help stabilization of RNA 40, 41, 42, 43.

The fluorescence intensities of specific signals for the egg and cheese samples were lower than those of other food samples. This might be the reason why the bacterial cells were not perfectly collected during pretreatment because the cells were located between the heavy and light layers of these samples. The hybridization result of the pork sample showed a high background intensity. The reason could be that the nonspecific binding of detector probes caused by the sticky components of meat were not separated during the preparation. Although complete RNAs directly obtained from food matrixes were used as target materials, the detection was successful because 16S rRNAs are abundant (10^4^–10^5^ per cell) in bacterial cells as actual target nucleic acids 44. The interferences by food matrixes were also reduced because most food particles could be removed simply using centrifugation.

Among pathogenic bacteria, 16 strains used in this study are important targets in the food industry and public hygiene, because they are major and frequent causes of outbreaks of foodborne disease 2, 45, 46, 47. Therefore, our geno‐biochip detection system could be a powerful solution to check for foodborne pathogen safety. Collectively, the geno‐biochip based on 16S rRNA information is able to selectively and practically detect multiple common bacterial pathogens in the food industry and public hygiene and would be useful to monitor food safety.

## Supporting information

As a service to our authors and readers, this journal provides supporting information supplied by the authors. Such materials are peer reviewed and may be re‐organized for online delivery, but are not copy‐edited or typeset. Technical support issues arising from supporting information (other than missing files) should be addressed to the authors.

Supporting Information

## References

[1] Newell, D. G. , Koopmans, M. , Verhoef, L. , Duizer, E. et al., Food‐borne diseases: The challenges of 20 years ago still persist while new ones continue to emerge. Int. J. Food Microbiol. 2010, 139 Suppl. 1, S3‐ S15. 20153070 10.1016/j.ijfoodmicro.2010.01.021 PMC7132498

[2] Korean Ministry of Food and Drug Safety, The Statistic System For Foodborne Disease. 2015.

[3] Linscott, A. J. , Food‐Borne Illnesses. Clin. Microbiol. Newsl. 2011, 33, 41–45.

[4] Clarridge, J. E. , 3rd, Impact of 16S rRNA gene sequence analysis for identification of bacteria on clinical microbiology and infectious diseases. Clin. Microbiol. Rev. 2004, 17, 840–862. 15489351 10.1128/CMR.17.4.840-862.2004 PMC523561

[5] Aytac, S. , Taban, B. , Food‐borne microbial diseases and control: Food‐borne infections and intoxications, in: Malik A., Erginkaya Z., Ahmad S., Erten H. (Eds.), Food Processing: Strategies for Quality Assessment, Springer New York 2014, pp. 191–224.

[6] Ge, B. , Meng, J. , Advanced technologies for pathogen and toxin detection in foods: Current applications and future directions. J. Lab. Autom. 2009, 14, 235–241.

[7] López‐Campos, G. , Martínez‐Suárez, J. , Aguado‐Urda, M. , López‐Alonso, V. , Detection, identification, and analysis of foodborne pathogens, in: Microarray Detection and Characterization of Bacterial Foodborne Pathogens, Springer US 2012, pp. 13–32.

[8] Hwang, B. H. , Lee, J. W. , Cha, H. J. , Polymerase chain reaction‐based detection of total and specific *Vibrio* species. Appl. Biochem. Biotechnol. 2010, 162, 1187–1194. 19937156 10.1007/s12010-009-8853-z

[9] Suo, B. , He, Y. , Tu, S. I. , Shi, X. , A multiplex real‐time polymerase chain reaction for simultaneous detection of *Salmonella* spp., *Escherichia coli* O157, and *Listeria monocytogenes* in meat products. Foodborne Pathog. Dis. 2010, 7, 619–628. 20113204 10.1089/fpd.2009.0430

[10] Ruiz‐Rueda, O. , Soler, M. , Calvo, L. , Garcia‐Gil, J. L. , Multiplex real‐time PCR for the simultaneous detection of *Salmonella* spp. and *Listeria monocytogenes* in food samples. Food Anal. Methods 2011, 4, 131–138.

[11] Ahn, K. , Lee, K. B. , Kim, Y. J. , Koo, Y. M. , Qunatitative analysis of the three main genera in effective microoraganisms using qPCR. Korean J. Chem. Eng. 2014, 31, 849–854.

[12] Zhang, D. , Xu, J. , He, W. , Tong, Q. , Chen, L. et al., Characterization of Enterobacter cloacae under phoxim stress by two‐dimensional gel electrophoresis. Biotechnol. Bioprocess Eng. 2015, 20, 403–409.

[13] Wagner, M. , Horn, M. , Daims, H. , Fluorescence in situ hybridisation for the identification and characterisation of prokaryotes. Curr. Opin. Microbiol. 2003, 6, 302–309. 12831908 10.1016/s1369-5274(03)00054-7

[14] Bisha, B. , Brehm‐Stecher, B. F. , Combination of adhesive‐tape‐based sampling and fluorescence in situ hybridization for rapid detection of Salmonella on fresh produce. J Visualized Exp. 2010, 14, 2308. 10.3791/2308 PMC3185619 21048665

[15] Hong, B. X. , Jiang, L. F. , Hu, Y. S. , Fang, D. Y. , Guo, H. Y. , Application of oligonucleotide array technology for the rapid detection of pathogenic bacteria of foodborne infections. J. Microbiol. Methods 2004, 58, 403–411. 15279944 10.1016/j.mimet.2004.05.005

[16] Eom, H. S. , Hwang, B. H. , Kim, D. H. , Lee, I. B. et al., Multiple detection of food‐borne pathogenic bacteria using a novel 16S rDNA‐based oligonucleotide signature chip. Biosens. Bioelectron. 2007, 22, 845–853. 16621503 10.1016/j.bios.2006.03.005

[17] Giannino, M. L. , Aliprandi, M. , Feligini, M. , Vanoni, L. et al., A DNA array based assay for the characterization of microbial community in raw milk. J. Microbiol. Methods 2009, 78, 181–188. 19482050 10.1016/j.mimet.2009.05.015

[18] Hwang, B. H. , Cha, H. J. , Pattern‐mapped multiple detection of 11 pathogenic bacteria using a 16s rDNA‐based oligonucleotide microarray. Biotechnol. Bioeng. 2010, 106, 183–192. 20091734 10.1002/bit.22674

[19] Hwang, B. H. , Shin, H. H. , Seo, J. H. , Cha, H. J. , Specific multiplex analysis of pathogens using a direct 16S rRNA hybridization in microarray system. Anal. Chem. 2012, 84, 4873–4879. 22551354 10.1021/ac300476k

[20] López‐Campos, G. , Martínez‐Suárez, J. , Aguado‐Urda, M. , López‐Alonso, V. , DNA microarrays: Principles and technologies, Microarray Detection and Characterization of Bacterial Foodborne Pathogens, Springer US 2012, pp. 33–47.

[21] Lane, D. J. , Pace, B. , Olsen, G. J. , Stahl, D. A. et al., Rapid determination of 16S ribosomal RNA sequences for phylogenetic analyses. Proc. Natl. Acad. Sci. USA 1985, 82, 6955–6959. 2413450 10.1073/pnas.82.20.6955 PMC391288

[22] Weisburg, W. G. , Barns, S. M. , Pelletier, D. A. , Lane, D. J. , 16S ribosomal DNA amplification for phylogenetic study. J. Bacteriol. 1991, 173, 697–703. 1987160 10.1128/jb.173.2.697-703.1991 PMC207061

[23] Suau, A. , Bonnet, R. , Sutren, M. , Godon, J. J. et al., Direct analysis of genes encoding 16S rRNA from complex communities reveals many novel molecular species within the human gut. Appl. Environ. Microbiol. 1999, 65, 4799–4807. 10543789 10.1128/aem.65.11.4799-4807.1999 PMC91647

[24] Kalinowski, R. M. , Tompkin, R. B. , Bodnaruk, P. W. , Pruett, W. P. , Jr., Impact of cooking, cooling, and subsequent refrigeration on the growth or survival of Clostridium perfringens in cooked meat and poultry products. J. Food Prot. 2003, 66, 1227–1232. 12870757 10.4315/0362-028x-66.7.1227

[25] Hwang, B. H. , Cha, H. J. , Quantitative oligonucleotide microarray data analysis with an artificial standard probe strategy. Biosens. Bioelectron. 2008, 23, 1738–1744. 18337080 10.1016/j.bios.2008.01.024

[26] Bowtell, D. , Sambrook, J. , DNA Microarrays, Cold Spring Harbor Laboratory Press 2003.

[27] Koehler, R. T. , Peyret, N. , Effects of DNA secondary structure on oligonucleotide probe binding efficiency. Comput. Biol. Chem. 2005, 29, 393–397. 16290040 10.1016/j.compbiolchem.2005.09.002

[28] Lane, S. , Evermann, J. , Loge, F. , Call, D. R. , Amplicon secondary structure prevents target hybridization to oligonucleotide microarrays. Biosens. Bioelectron. 2004, 20, 728–735. 15522587 10.1016/j.bios.2004.04.014

[29] Hagan, M. F. , Chakraborty, A. K. , Hybridization dynamics of surface immobilized DNA. J. Chem. Phys. 2004, 120, 4958–4968. 15267358 10.1063/1.1645786

[30] Wang, X. , Son, A. , Effects of pretreatment on the denaturation and fragmentation of genomic DNA for DNA hybridization. Environ. Sci. Processes Impacts 2013, 15, 2204–2212. 10.1039/c3em00457k 24162665

[31] Jorgensen, J. H. , Pfaller, M. A. , Carroll, K. C. , Funke, G. et al., Manual of Clinical Microbiology, 11th Edition, American Society of Microbiology 2015.

[32] Lan, R. , Reeves, P. R. , *Escherichia coli* in disguise: Molecular origins of *Shigella* . Microbes Infect. 2002, 4, 1125–1132. 12361912 10.1016/s1286-4579(02)01637-4

[33] Fukushima, M. , Kakinuma, K. , Kawaguchi, R. , Phylogenetic analysis of *Salmonella*, *Shigella*, and *Escherichia coli* strains on the basis of the gyrB gene sequence. J. Clin. Microbiol. 2002, 40, 2779–2785. 12149329 10.1128/JCM.40.8.2779-2785.2002 PMC120687

[34] Christensen, H. , Nordentoft, S. , Olsen, J. E. , Phylogenetic relationships of *Salmonella* based on rRNA sequences. Int. J. Syst. Bacteriol. 1998, 48 Pt. 2, 605–610. 9731304 10.1099/00207713-48-2-605

[35] Shin, H. H. , Hwang, B. H. , Seo, J. H. , Cha, H. J. , Specific discrimination of three pathogenic *Salmonella enterica* subsp. *enterica* serotypes by *carB*‐based oligonucleotide microarray. Appl. Environ. Microbiol. 2014, 80, 366–373. 24185846 10.1128/AEM.02978-13 PMC3911022

[36] Cremonesi, P. , Pisoni, G. , Severgnini, M. , Consolandi, C. et al., Pathogen detection in milk samples by ligation detection reaction‐mediated universal array method. J. Dairy Sci. 2009, 92, 3027–3039. 19528580 10.3168/jds.2008-1773

[37] Shin, H. H. , Seo, J. H. , Kim, C. S. , Hwang, B. H. et al., Hybrid microarray based on double biomolecular markers of DNA and carbohydrate for simultaneous genotypic and phenotypic detection of cholera toxin‐producing *Vibrio cholerae* . Biosens. Bioelectron. 2016, 79, 398–405. 26735874 10.1016/j.bios.2015.12.073

[38] Srinivasan, J. , Miller, J. , Kollman, P. A. , Case, D. A. , Continuum solvent studies of the stability of RNA hairpin loops and helices. J. Biomol. Struct. Dyn. 1998, 16, 671–682. 10052623 10.1080/07391102.1998.10508279

[39] Diaz, E. , Barisone, G. A. , DNA microarrays: Sample quality control, array hybridization and scanning. J. Visualized Exp. 2011, 49, 2546. 10.3791/2546 PMC3197308 21445042

[40] Misra, V. K. , Draper, D. E. , On the role of magnesium ions in RNA stability. Biopolymers 1998, 48, 113–135. 10333741 10.1002/(SICI)1097-0282(1998)48:2<113::AID-BIP3>3.0.CO;2-Y

[41] Laing, L. G. , Gluick, T. C. , Draper, D. E. , Stabilization of RNA structure by Mg ions: Specific and non‐specific effects. J. Mol. Biol. 1994, 237, 577–587. 8158638 10.1006/jmbi.1994.1256

[42] Park, M. K. , Kim, K. W. , Ahn, D. J. , Oh, M. K. , Label‐free detection of bacterial RNA using polydiacetylene‐based biochip. Biosens. Bioelectron. 2012, 35, 44–49. 22410489 10.1016/j.bios.2012.01.043

[43] Blacksell, S. D. , Khounsy, S. , Westbury, H. A. , The effect of sample degradation and RNA stabilization on classical swine fever virus RT–PCR and ELISA methods. J. Virol. Methods 2004, 118, 33–37. 15158066 10.1016/j.jviromet.2004.01.015

[44] Zwiglmaier, K. , Ludwig, W. , Schleifer, K. H. , Recognition of individual genes in a single bacteria cell by fluorescence in situ hybridization‐RING‐FISH. Mol. Microbiol. 2004, 51, 89–96. 14651613 10.1046/j.1365-2958.2003.03834.x

[45] Centers for Disease Control and Prevention (CDC), *Surveillance for Foodborne Disease Outbreaks, United States, 2012* , Annual Report, CDC, Atlanta, Georga, USA 2014.

[46] European Food Safety Authority (EFSA), The European Union summary report on trends and sources of zoonoses, zoonotic agents and food‐borne outbreaks in 2013. EFSA J. 2015, 13, 3991. 10.2903/j.efsa.2018.5500 PMC7009540 32625785

[47] Demirci, A. , Ngadi, M. O. , Microbial Decontamination in the Food Industry: Novel Methods and Applications, Elsevier Science 2012.

