# Supplementary material for: Multiplex 16S rRNA‐derived geno‐biochip for detection of 16 bacterial pathogens from contaminated foods
Source: Biotechnol J. 2016 Sep 6;11(11):1405–14. doi: 10.1002/biot.201600043 (PMC5132059; doi:10.1002/biot.201600043)
Supplement: Supplementary file 1 — Supporting Information [file BIOT-11-1405-s001.pdf]

Biotechnology
Journal

Supporting Information for DOI 10.1002/biot.201600043

Multiplex 16S rRNA-derived geno-biochip for detection of 16 bacterial pathogens from contaminated foods

Hwa Hui Shin, Byeong Hee Hwang, and Hyung Joon Cha

© 2016 Wiley-VCH Verlag GmbH & Co. KGaA, Weinheim

Supporting information

Multiplex 16S rRNA-derived geno-biochip for detection of 16 bacterial pathogens from contaminated foods

Hwa Hui Shin[1,+], Byeong Hee Hwang[1,2,+], Hyung Joon Cha[1,*]

[1]Department of Chemical Engineering, Pohang University of Science and Technology, Pohang, 790-784, Korea

[2]Division of Bioengineering, Incheon National University, Incheon 406-772, Korea

[+]Equal contribution

Correspondence: Prof. Hyung Joon Cha, Department of Chemical Engineering, Pohang University of Science and Technology, 790-784, Korea

E-mail: hjcha@postech.ac.kr

Table S1. Bacterial strains used in this study.

Bacteria	Source and strain	Biosafety level
Bacillus cereus	ATCC 11778	1
	ATCC 13061	1
	ATCC 14579	1
Campylobacter jejuni	ATCC 33291	2
Clostridium perfringens	ATCC 13124	2
Escherichia coli	ATCC 25922	1
Escherichia coli O157:H7	ATCC 43894	2
Listeria monocytogenes	ATCC 15313	2
Salmonella enterica subsp. *enterica* serotype Choleraesuis	ATCC 10708	2
	ATCC 13312	2
	ATCC 7001	2
Salmonella enterica subsp. *enterica* serotype Enteritidis	ATCC 31194 (IFO 3313)	2
Shigella boydii	ATCC 8700	2
	ATCC 35966	2
Shigella dysenteriae	ATCC 13313	2
Shigella sonnei	ATCC 9290	2
	ATCC 25931	2
	ATCC 29930	2
Staphylococcus aureus	ATCC 6538	2
Vibrio cholerae	ATCC 14035	2
Vibrio parahaemolyticus	ATCC 17802	2
Vibrio vulnificus	ATCC 27562	2
Yersinia enterocolitica	ATCC 23715	2

ATCC: American Type Culture Collection (Manassas, VA, USA).
IFO: Institute for Fermentation (IFO; Osaka, Japan)

Table S2. Newly designed specific capture probes and their thermodynamic properties[a].

Bacteria	Probe Name	Sequences (5'-3', 5'-amine-spacer, spacer:C6)	Length (bp)	T_m (°C)	Rating
Bacillus cereus	BACE-1	CCTCGCGGTCTTGCAGCTCTT	20	63.0	81
	BACE-2	CCACCTGTCACTCTGCTCCCG	21	65.0	100
	BACE-3	CTGCTCCCGAAGGAGAAGCC	20	63.6	85
Campylobacter jejuni	CAJE-1	CACTCTAGACTATCAGTTTCCCAAGC	26	68.6	88
	CAJE-2	TACCCCTACACCACCAATTCCATCTG	26	67.3	91
Clostridium perfringens	CLPE-1	CGGAGGTGTTGAAACCCCCA	20	65.0	100
	CLPE-2	CACCCCAATCGCTGACCCTA	20	62.9	100
Shigella boydii	SHBO-1	CCCCACCAACAAGCTAATCCC	21	63.1	57
	SHBO-2	ACATTCTCATCTCTGAAAACTTCCGT	26	62.1	92
	SHBO-3	GCGAATCAGCAAGCTGATTCC	21	62.3	68
Shigella sonnei	SHSO-1	GCGAAACAGCAAGCTGTTTCC	21	62.5	67
	SHSO-2	ACCAATCCATCTCTGGAAAGTTCTGT	25	62.9	86

[a] All thermodynamic properties were calculated by Primer Premier.

Table S3. Detection results from Figure S2 for 6 pathogenic bacteria.

**Bacteria**	**Detection results**		
	**Positive[a]**		**Negative[b]**
	**True**	**False**	**False**
B. cereus	BACE-1,2, and 3	STAU-1	
C. perfringens	CLPE-1		CLPE-2
C. jejuni	CAJE-1 and 2	BACE-3 and STAU-1	
S. boydii	SHBO-1 and 2	ESCO, SHDY-2, and SHSO-1	SHBO-3
S. dysenteriae	SHDY-1 and 2	ESCOO-2 and SHSO-1	
S. sonnei	SHSO-1	BACE-3, SHBO-2, and SHDY-2	SHSO-2

[a] Positive if $S/N \geqq 2$

[b] Negative if $S/N < 2$

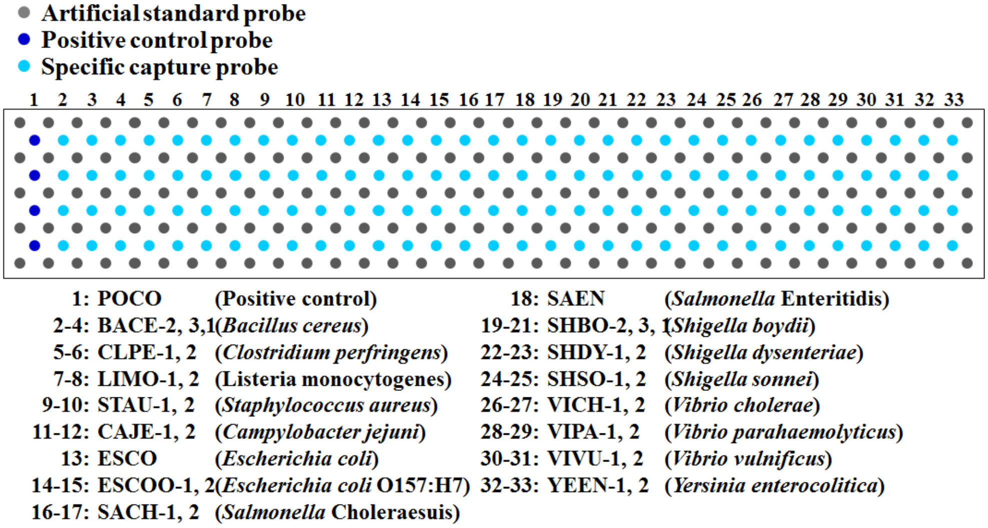

Figure S1. The schematic diagrams of the array format for the 16S rRNA-derived geno-biochip containing 32 specific DNA oligonucleotide capture probes before the final selection

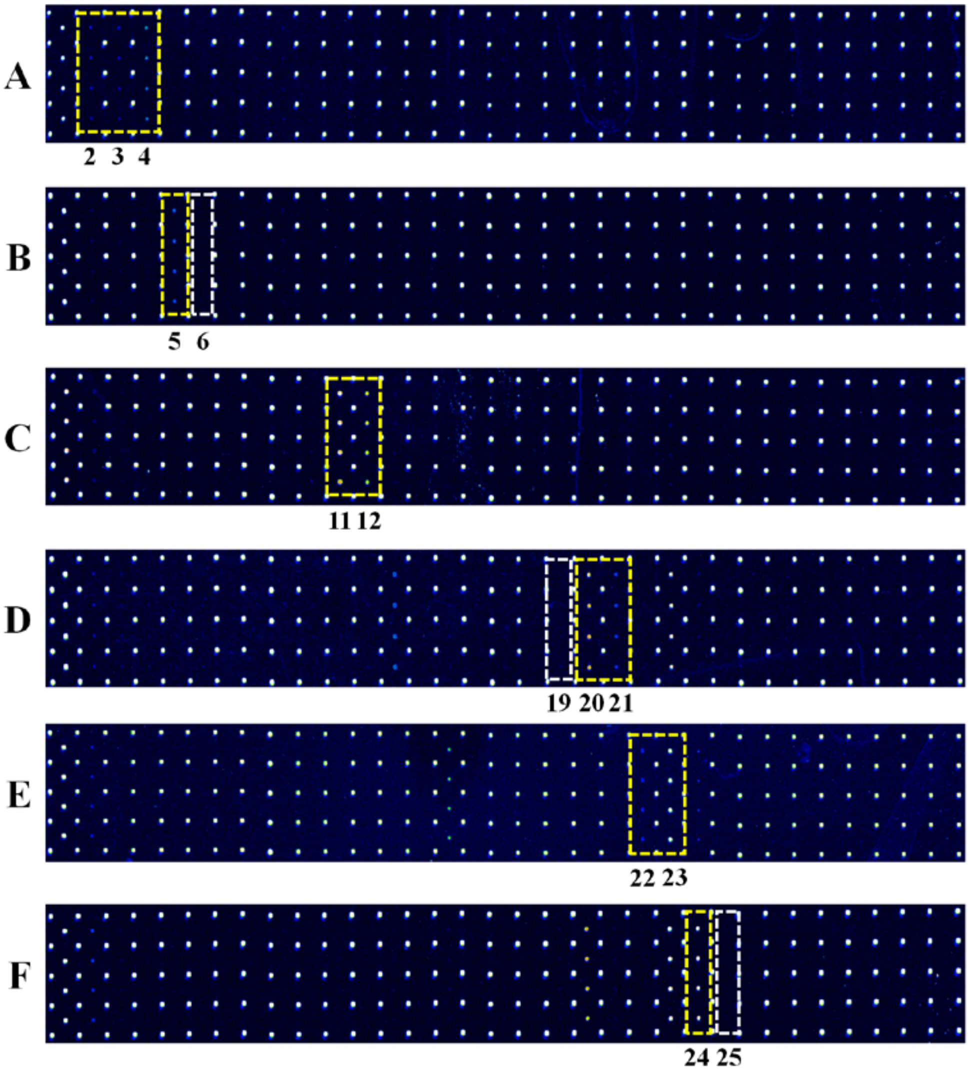

Figure S2. The raw images of hybridization with each amplified 16S rDNA target using the array format in Figure S1: (A) *B. cereus*, (B) *C. perfringens*, (C) *C. jejuni*, (D) *S. boydii*, (E) *S. dysenteriae*, and (F) *S. sonnei*. The yellow dotted boxes indicate observed specific spots and the white dotted boxes indicate unobserved specific spots. The numbers indicate those of capture probes in the array format.

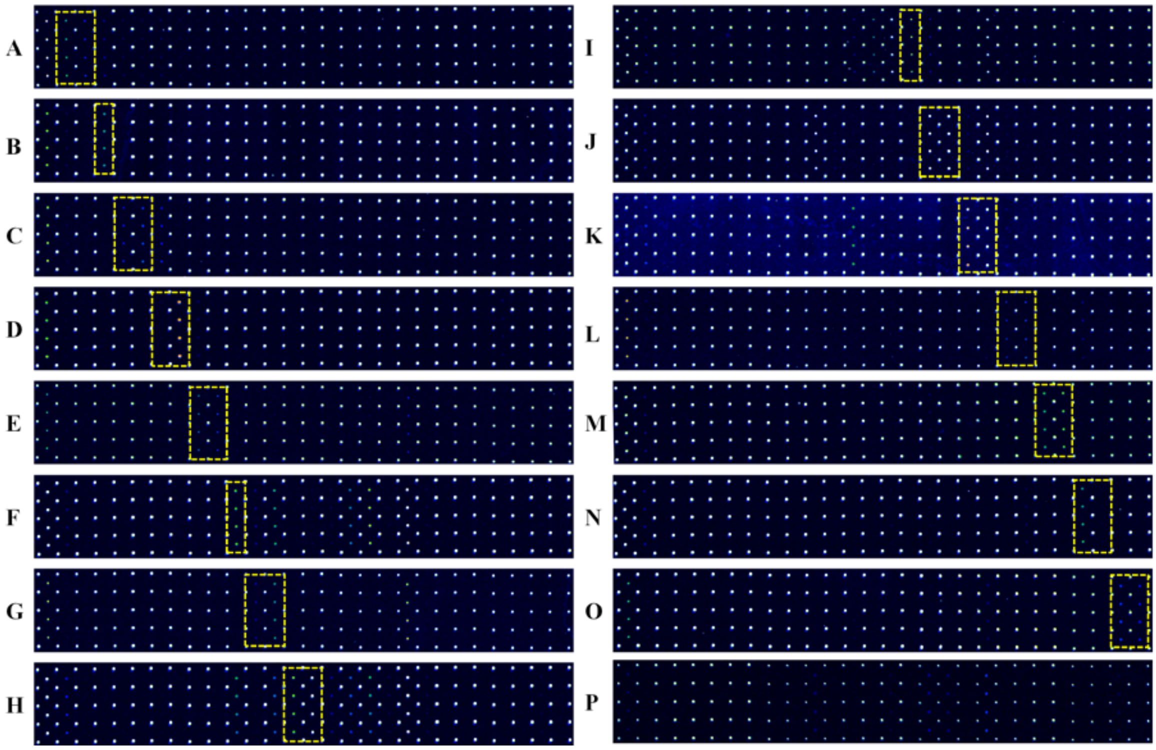

Figure S3. The raw images of hybridization with each amplified 16S rDNA target using the array format in Figure 1: (A) *B. cereus*, (B) *C. perfringens*, (C) *L. monocytogenes*, (D) *S. aureus*, (E) *C. jejuni*, (F) *E. coli*, (G) *E. coli* O157:H7, (H) *S. enterica* subsp. *enterica* serotype Choleraesuis (I) *S. enterica* subsp. *enterica* serotype Enteritidis (J) *S. boydii*, (K) *S. dysenteriae*, (L) *V. cholerae*, (M) *V. parahaemolyticus*, (N) *V. vulnificus*, (O) *Y. enterocolitica*, *and* (P) *S. sonnei*. The yellow dotted boxes indicate observed specific spots.

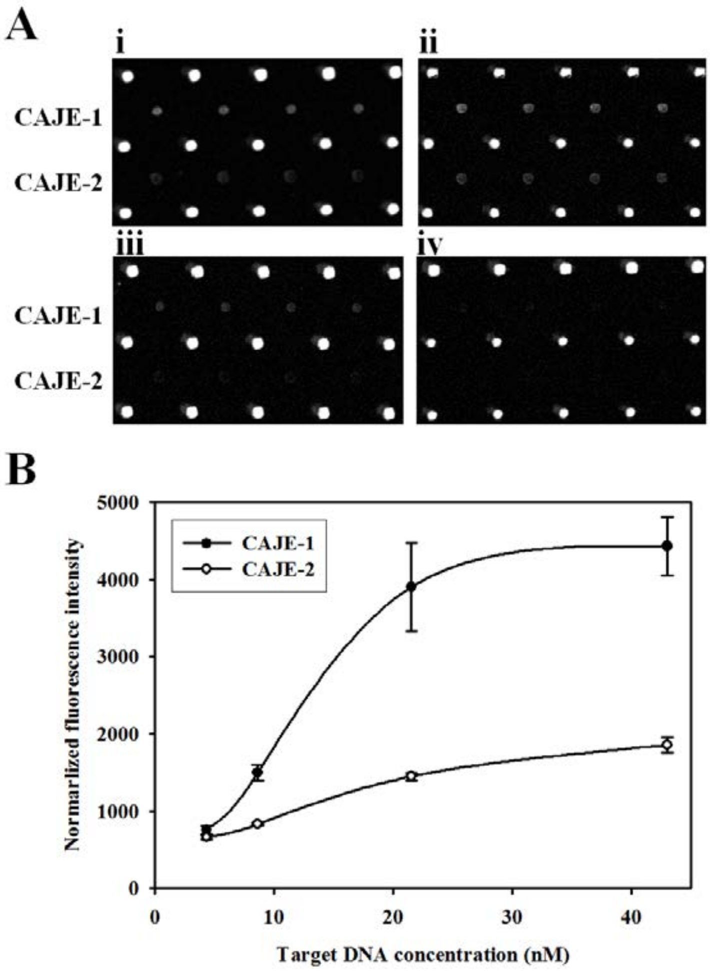

Figure S4. Sensitivity of the 16S rRNA-derived geno-biochip using *C. jejuni*. The diluted PCR amplicons were used for sensitivity determination. (A) Raw hybridization images for (i) 43 nM, (ii) 21.5 nM, (iii) 8.6 nM, and (iv) 4.3 nM 16S rDNA targets; and (B) the plot of dynamic detection ranges based on the normalized fluorescence intensities according to target DNA concentration changes for each capture probe CAJE 1 (closed circle) and CAJE 2 (open circle). Each value was the mean of 4 repeated spots, and the error bars represent standard deviation.
